# Real-Time Muscle Activity and Joint Range of Motion Monitor to Improve Shoulder Pain Rehabilitation in Wheelchair Basketball Players: A Non-Randomized Clinical Study

**DOI:** 10.3390/clinpract12060111

**Published:** 2022-12-16

**Authors:** Giacomo Farì, Marisa Megna, Pietro Fiore, Maurizio Ranieri, Riccardo Marvulli, Valerio Bonavolontà, Francesco Paolo Bianchi, Filomena Puntillo, Giustino Varrassi, Victor Machado Reis

**Affiliations:** 1 Department of Translational Biomedicine and Neuroscience (DiBraiN), Aldo Moro University, 70121 Bari, Italy; 2 Department of Biological and Environmental Science and Technologies (Di.S.Te.B.A.), University of Salento, 73100 Lecce, Italy; 3 Istituti Clinici Scientifici Maugeri, IRCCS Institute of Bari, 70121 Bari, Italy; 4 Department of Biotechnological and Applied Clinical Sciences, University of L’Aquila Vetoio, 67100 L’Aquila, Italy; 5 Department of Interdisciplinary Medicine, Aldo Moro University of Bari, 70121 Bari, Italy; 6 Paolo Procacci Foundation, 00193 Rome, Italy; 7 Research Centre in Sport Sciences, Health Sciences and Human Development, 5001-801 Vila Real, Portugal

**Keywords:** rehabilitation, outcome, wheelchair, basketball, sport, Paralympics, shoulder, electromyography, adaptive sports, disability, biomechanics

## Abstract

Wheelchair basketball (WB) involves sports gestures that expose the shoulder to high biomechanical stress and frequently lead to shoulder pain (SP). Due to their physical peculiarities and sporting performance, these athletes require specific rehabilitation programs that are as fast, personalized and effective as possible. However, there are few studies specifically dedicated to these purposes. Surface electromyography (sEMG) seems a promising tool for better customization and achieving more targeted rehabilitation results. The aim of this study was to evaluate the usefulness of sEMG to monitor SP rehabilitation outcomes in WB players. Thirty-three athletes were enrolled in this non-randomized clinical study and divided into two groups. Both groups underwent a shoulder rehabilitation protocol, but only the experimental group was monitored in real time with sEMG on the shoulders. At enrollment (T0), at the end of 4 weeks of the rehabilitation program (T1), and 8 weeks after T1 (T2), the following outcome measures were collected: Wheelchair User’s Shoulder Pain Index (WUSPI), 20 m straight line test, shoulder abduction range of motion (ROM). There was a statistically significant difference for WUSPI and ROM scores in the comparison between groups (*p* < 0.001), and for all outcomes in the comparison between times and in the interaction between time and group (*p* < 0.001). Therefore, the experimental group showed a better improvement at all detection times compared to the control group. sEMG seems a useful tool for improving the monitoring of SP rehabilitation outcomes in WB players. This monitoring speeds up and improves the rehabilitative results, limiting the risk of sport abandonment and increasing the possibility for people with disabilities to quickly return to practice physical activity.

## 1. Introduction

Wheelchair basketball (WB) is a variation of basketball that is played by people with different physical disabilities. Born in the USA in the late 1940s as a rehabilitation activity for veterans of World War II, it quickly grew in popularity, becoming a Paralympic sport in the edition held in Rome in 1960 [1]. WB grants many physical and psychological benefits for people with disabilities who practice it [2]. Moreover, it represents a great occasion to break down the barriers that limit social inclusion that often result from the condition of disability [3].

However, WB is characterized by sports gestures that require high physical performance and therefore expose players to the risk of injuries and musculoskeletal diseases. A recent systematic review found that shoulder is the body region most affected by this sport related injuries [4]. In fact, WB requires athletes to perform rapid wheelchair propulsion movements aimed at sprinting and changing direction on the court, as well as repeated ball throws for passes and shots. As a consequence, shoulders are constantly stressed in rapid rotational and abduction movements and are particularly exposed to overload injuries that cause shoulder pain (SP) [5,6]. Karasuyama et al. investigated SP related to WB and estimated a prevalence ranging from 38% to 75% [7]. SP therefore risks leading to the suspension or abandonment of sporting activity in those who practice WB at all levels. Moreover, SP limits wheelchair users in activities related to daily living, becoming more dependent on others, especially in transfers. Therefore, prevention and rehabilitation of SP in WB players are an ever-present challenge in sports medicine [8]. WB players cannot be trivially equated to other categories of sportsmen, and SP in these athletes requires specific therapeutic and rehabilitative paths. Despite this, there are few studies specifically dedicated to these purposes [9,10]. Although these studies are valuable for the growing of knowledge in this particular sector, further studies are needed to investigate the use of new rehabilitation tools, more objective outcome measures, and more quickly effective rehabilitation programs.

New technologies are becoming increasingly available in sport medicine and rehabilitation. Surface electromyography (sEMG) is a traditional diagnostic instrument that in recent years has also been used for monitoring the execution of therapeutic exercise [11,12,13,14]. In this sense, sEMG seems a promising tool for providing an objective assessment of sports rehabilitation programs results, but there is still a lack of evidence regarding the possibility that it could better customize and speed up these results, especially for athletes with specific characteristics and equally specific needs such as those who practice WB.

The aim of this study was to evaluate the usefulness of sEMG to monitor SP rehabilitation outcomes in WB players in real time. We assume that this biofeedback application could speed up traditional rehabilitation programs, reducing pain and improving joint function faster and more effectively.

## 2. Materials and Methods

A prospective non-randomized clinical study was conducted at the Movement Analysis Service of the Department of Biological and Environmental Science and Technology, University of Salento, Lecce, Italy between May 2021 and September 2022.

WB athletes currently playing in the second Italian division of FIPIC (Wheelchair Basketball Italian Federation) were eligible for recruitment, provided that they met the following inclusion criteria: age > 18 years; membership in a professional WB sports association; sporting practice for at least 2 years; SP for at least 1 month; medical and ultrasound diagnosis of rotator cuff and/or biceps tendinopathy and/or contractures of the scapulohumeral girdle muscles; at least 5 years of prevalent wheelchair use in the activities of daily life (i.e., that wheelchair was used for all the main activities of daily life and therefore for almost the whole day). Exclusion criteria: SP treatments (e.g., physiotherapy, injections, surgery) in the previous month; presence of shoulder fractures and arthropathies, presence of complete rotator cuff tendons tears, clinical or instrumental evidence of rheumatological or neurological diseases affecting the upper limbs.

Thirty-three (33) athletes met these criteria and were recruited for the study. The sample size was a convenience one, but it was in line with previous studies concerning biofeedback and robotic interventions [15,16,17]. Moreover, G. Power post hoc calculations for the ANOVA that was performed indicated a statistical power of 96%, provided a minimum effect size of 0.3 (as given by eta square).

At enrolment (T0), each athlete underwent a medical evaluation and was subjected to a battery of tests as described below. Then, all the recruited WB players were divided into two groups: experimental group and control group. Both groups underwent a shoulder rehabilitation protocol under the guidance of a therapist according to an exercise protocol for wheelchair sport athlete with SP [18] (four weeks, two sessions per week, 1 h per session). Exercises were executed in a rehabilitation gym and were adapted for each individual within a pain-free range and focused on the stretching and strengthening of shoulder rotators, adductors, abductors and extensors, with particular attention to deltoids. The difference between the groups consisted of the fact that subjects in the experimental group executed all the therapeutic exercises under the control of mDurance^®^ system, which made it possible for therapist and athlete to monitor in real time the activity of anterior and posterior deltoid muscles of both shoulders and the shoulder range of motion (ROM). This system (mDurance Solutions SL, Granada, Spain) is a portable sEMG that consists of different parts. A Shimmer EMG unit (Realtime Technologies Ltd., Dublin, Ireland) consisting of a bipolar sensor was used for the recording of superficial muscle activity. Each sensor has two channels, with a sampling rate of 1024 Hertz and a signal resolution of 24 bits. The electrodes are pre-gelled and have a diameter of 10 mm and an inter-electrode distance of 20 mm. A dedicated mobile application is responsible for receiving data from the Shimmer unit and sending it to a cloud service, where signals are stored and analyzed, making them visible in real time on a tablet screen and obtaining the final reports [13]. In this way, a therapist guided the execution of the exercises by checking the muscular activity of the deltoids and the joint mobility on a screen, while at the same time the biofeedback WB player corrected the execution of the exercises.

Each enrolled subject was newly evaluated at T1, at the end of the rehabilitation protocol, 4 weeks after T0, and at T2, 8 weeks after T1. These evaluations included the following tests and scales:

- WUSPI (Wheelchair User’s Shoulder Pain Index): this is a scale that measures shoulder pain associated with the functional activities of wheelchair users. This 15-item functional investigates shoulder pain during transfers, self-care, wheelchair mobility and general activities. The score can range from 0 to 150 [19].
- Twenty meter straight line test: this is an instrument for wheelchair speed evaluation [20]. At the starting signal the athlete sprints, covering a 20 m distance on a straight line in the shortest time possible. Two attempts are given, and just the best one is recorded.
- Range of motion (ROM) in abduction: this is the evaluation measured in degrees° of the shoulder range of movement in the direction most influenced by the activity of the deltoid muscle. This evaluation was performed using the inertial sensors included in the mDurance device.

In the twelve weeks after T0, athletes were allowed to take paracetamol as needed (maximum 3 g/day) and were asked to report the frequency and dosage using an intake diary.

Ethical approval was granted by the Institutional Review Board of University of Salento (n.3/28.04.2021). All the procedures were carried out in accordance with the principles of the Helsinki Declaration. Each participant was recruited with informed consent to participate in the study.

### Statistical Analysis

Compiled forms were entered into a database created using an Excel spreadsheet, and data analysis was performed using Stata MP17 software. Continuous variables are described as mean ± standard deviation (SD) and range, and categorical variables are described as proportions. The skewness and kurtosis test was used to evaluate the normality of continuous variables; all the continuous variables were normally distributed. Student’s *t*-test for independent data was used to compare continuous variables between groups, and the ANOVA for repeated measures test was used to compare continuous variables between groups and detection times; a post hoc analysis was performed using the test of simple effects to estimate the variation of each outcome confronting each detection time per group. The chi-square test was used to compare the proportions between groups. For all tests, a two-sided *p*-value < 0.05 was considered statistically significant.

## 3. Results

The study sample was made up of 33 spinal-cord-injured male subjects, of which 17 (51.5%) belonged to the control group and 16 (48.5%) to the experimental group; the characteristics of the sample, by group, are shown in Table 1. The groups were homogeneous on the basis of the considered variables.

**Figure 1:**
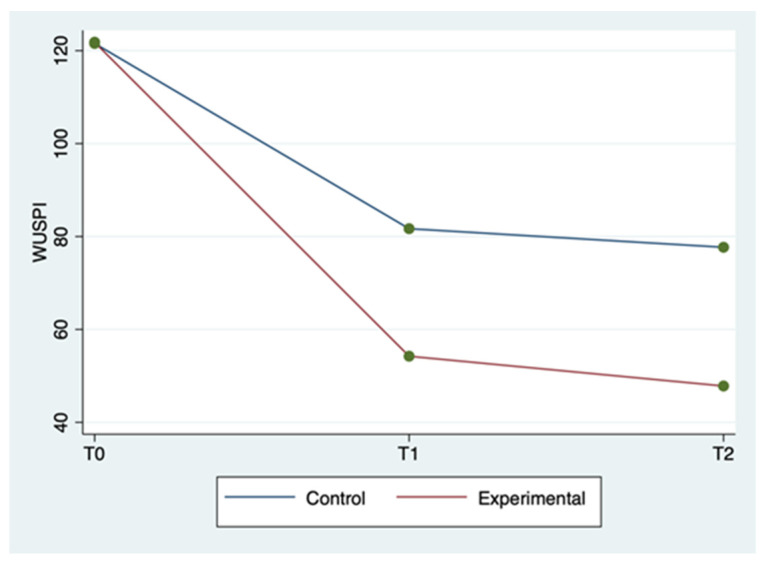
WUSPI mean score by group and detection time.

**Figure 2:**
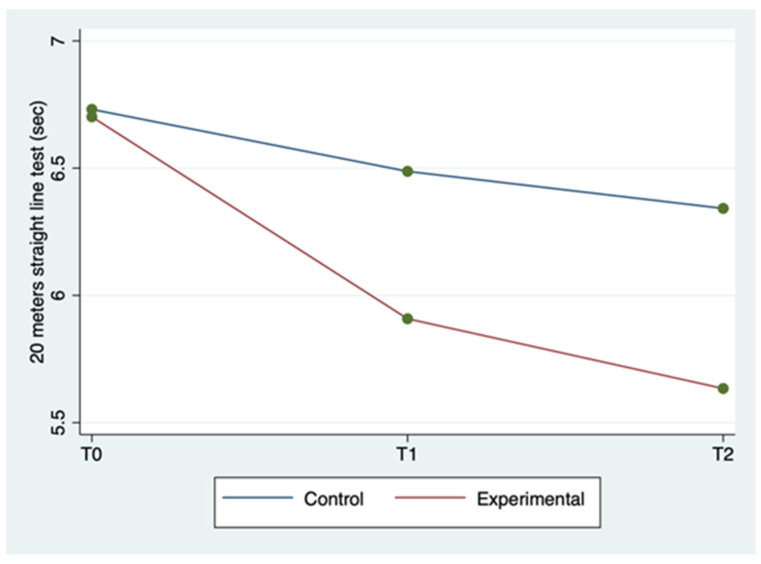
Twenty meter straight line test mean score by group and detection time.

**Figure 3:**
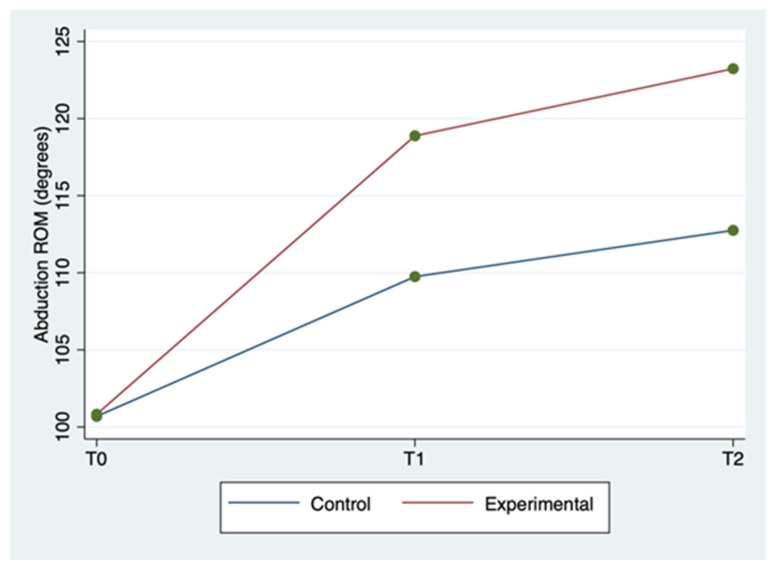
Shoulder abduction ROM mean score by group and detection time.

The mean ± SD and range of the outcome variables, by group and time of detection, are described in Table 2. WUSPI scores improved in both groups between the three detection times, but more markedly in the experimental group (Figure 1). The 20 m straight line test scores improved in the experimental group between the three detection times, while for the control group, they improved less markedly and only between T0 and T1, remaining constant between T1 and T2 (Figure 2). Abduction ROM scores improved in both groups between the three detection times, but more markedly in the experimental group (Figure 3).

The ANOVA for repeated measures tests showed a statistically significant difference for WUSPI and abduction ROM scores in the comparison between groups (*p* < 0.0001) and for all of the outcome measures in the comparison between times (*p* < 0.0001). The same test showed a statistically significant difference for all the outcome measures in the interaction between time and group (*p* < 0.0001). Therefore, the experimental group showed a better improvement for all outcomes in terms of detection time compared to the control group. All these findings are described in Table 2.

In Table 3, a statistically significant improvement in the WUSPI scores emerged for both groups between T0 and T1, between T0 and T2, and between T1 and T2 (*p* < 0.05). A statistically significant improvement in the 20 m straight line test scores also emerged for both groups between T0 and T2, and between T0 and T2 (*p* < 0.05), while only the experimental group improved between T1 and T2 (*p* = 0.002). The abduction ROM scores improved in both groups between T0 and T1, between T0 and T2, and between T1 and T2 (*p* < 0.05). 

From the analysis of analgesic intake diaries in the twelve weeks following the enrollment, only a random intake emerged, which settled on an average of 1.5 g/week per group, with a sporadic and not significant distribution among the participants. 

## 4. Discussion

The aim of this study was to explore the usefulness of sEMG to monitor, better target, and improve SP rehabilitation and related outcomes in WB players. In fact, shoulder injuries are frequent in many sports [21,22], but they are even more frequent in wheelchair sports [23]. As a consequence, SP is one of the most common complaints for these sportsmen, particularly among WB players, who show the highest intensity performance for the upper extremities among wheelchair athletes [24]. Moreover, every possible therapy collides with the impossibility of complete rest, since the upper limbs are necessary for activities of daily living such as common movements [25]. It follows that treatments have to be targeted, specific and included in a personalized rehabilitation program [26]. A systematic review by Cratsenberg et al. [27] supported the effectiveness of various rehabilitative interventions in the management and treatment of SP, as demonstrated by a long-term but slow reduction in self-reported WUSPI scores. Therapeutic exercise is feasible, but there is a need to develop a standardized set of exercises that can be customized to the specific characteristics of wheelchair users and that can be faster to receive benefits [27]. Moreover, there is a lack in terms of functional outcomes assessment as these are often self-reported, so it is more difficult to achieve both the correct control of rehabilitation treatments and a more satisfactory recovery [28,29,30,31]. In this sense, sEMG could represent a great opportunity to refine the assessments available to rehabilitators. It is a safe and valid tool that is potentially capable of improving and accelerating the results of rehabilitation treatments, since it guarantees the real-time monitoring of muscle activity and joint range of motion [15]. 

The above-mentioned findings seem to show that the experimental group obtained better results for all outcome measures in all of the detection times compared to the control group, as described in Table 2 and Table 3. In particular, both groups improved between T0 and T2, but the experimental group in a more rapid and marked way. Thus, sEMG is effective in speeding up shoulder rehabilitation. In fact, this resulted in better restoration of the global joint functionality by significantly reducing SP, as evidenced by the WUSPI improvements. These findings are in line with those reported in other studies concerning therapeutic exercise programs for shoulder pain in manual wheelchair users [32,33]. In particular, Middaugh et al. [33] investigated the effect of EMG biofeedback training, in addition to a standard exercise program, on reducing SP in manual wheelchair users with spinal cord injury. They found that SP, as measured by WUSPI, decreased by 64% betwen baseline adn 10-week follow up in the EMG Biofeedback plus Exercise group, whereas at the same detection time in the Exercise group, it decreased by 27%. These results are comparable with our results at T2 (12 weeks after baseline), at which point WUSPI score had decreased by 61.7% in the experimental group and 36.1% in control group. As a result, adding sEMG to exercise protocols made the latter both faster and better aimed toward the functional needs of wheelchair users. Hence, the obtained abduction ROM increase is the logical and foreseeable consequence of a SP reduction and results in an improvement in overall joint function [28]. Furthermore, the sport-specific functionality increases, as demonstrated by the improvements in the 20 m straight line test. Riley et al. [18] provided a specific shoulder rehabilitation protocol for wheelchair athletes affected by SP. The authors stated that shoulder injuries rehabilitation and prevention have to be boosted by using clinical task analysis of shoulder biomechanics during wheelchair sport movement patterns. sEMG seems a great instrument to do that, since it increases the muscles’ activity control. In fact, it could allow the physiotherapist to correct in real time the execution of therapeutic exercise monitoring constantly articular ROM and electrical activity of the muscles of both shoulders. Consequently, the joint movements are made harmonious and synchronic and the dyskinesias are rapidly countered. Moreover, sEMG is simple to use, since mDurance provides a step-by-step guide that practically explains and graphically represents on the tablet screen all the procedures, from applying the sensors to reading the final results. Therefore, sEMG could provide the opportunity to improve the effectiveness of home-based shoulder rehabilitation protocols, whose current main limitation is the difficulty of carrying out the exercises correctly, and therefore by the slowness in achieving the relative benefits [34,35,36,37]. 

Finally, the opportunity to speed up and improve the rehabilitative results using sEMG could limit the risk of sports prolonged suspension or abandonment for WB players, safeguarding regular and safe sporting practice as an opportunity for psycho-physical health for people with disabilities.

This study has some limitations. First of all, the sample was a convenience one due to the fact that WB players are a small cohort in and of themselves. However, this sample is in line with other studies on the same matter, as stated in the method section, and it enabled a statistical power of 96%. The follow-up period was short, and further research is needed to establish whether the obtained results remain constant over the long term. However, the purpose of our study was to understand if sEMG was able to monitor and improve rehabilitation outcomes, and to do this it was important first of all to verify the immediate effectiveness of this tool, since the recovery needs of athletes are just as immediate.

Thus, further studies should test the effectiveness of sEMG as a real-time monitor for sport rehabilitation, especially in wheelchair athletes. Larger cohorts, longer follow-up periods, and a greater number of randomized clinical trials are needed to reinforce the scope of the current findings. However, sEMG seems a promising tool to better objectify, standardize and improve rehabilitation outcomes for wheelchair athletes, and it could be extended to many areas of sports rehabilitation.

## 5. Conclusions

The usefulness of sEMG lies in the possibility that this tool makes it possible to better monitor and target SP rehabilitation outcomes in WB players. It speeds up and improves the rehabilitative results, and in this way it limits the risk of sports prolonged suspension or abandonment. Moreover, a more rapid return to the field can also translate into a more effective restoration of motor autonomy in athletes who habitually use the wheelchair. Further studies and larger WB players cohorts are needed to deepen the effectiveness of sEMG as a real-time monitor for sport rehabilitation protocols, especially in wheelchair athletes, in order to increase the possibility for people with disabilities to play sports and therefore to improve their psychophysical health.

## Figures and Tables

**Table 1:** Sample features, by group.

Variable	Control (*n =* 16)	Experimental (*n =* 17)	Total (*n =* 33)	*p*-Value
Age; mean ± SD (range)	38.7 ± 8.7 (23–55)	37.2 ± 10.0 (24–57)	37.9 ± 9.3 (23–57)	0.648
BMI; mean ± SD (range)	23.7 ± 3.2 (18.8–31.1)	24.4 ± 4.9 (17.0–35.9)	24.0 ± 4.1 (17.0–35.9)	0.623
Right dominant limb; *n* (%)	13 (81.3)	12 (70.6)	25 (75.8)	0.475
Shoulder pain on the right side; *n* (%)	14 (87.5)	13 (76.5)	27 (81.8)	0.412

Control = control group; Experimental = experimental group; BMI = body mass index; SD = standard deviation; *n =* number.

**Table 2:** Mean ± Standard Deviation (range) of the outcomes, by group and detection time.

Outcome	Group	T0	T1	T2	Comparison between Groups	Comparison between Times	Interaction between Time and Group
WUSPI	Control	121.6 ± 9.6 (107–137)	81.7 ± 6.3 (71–92)	77.7 ± 6.6 (66–89)	<0.001	<0.001	<0.001
	Experimental	121.8 ± 8.9 (106–135)	54.2 ± 8.5 (41–70)	47.8 ± 8.7 (36–67)			
	Total	121.7 ± 9.1 (106–137)	67.5 ± 15.8 (41–92)	62.3 ± 17.0 (36–89)			
20 m straight line test (seconds)	Control	6.7 ± 0.6 (5.7–7.7)	6.5 ± 0.6 (5.6–7.5)	6.5 ± 0.6 (5.6–7.5)	0.014	<0.001	<0.001
	Experimental	6.7 ± 0.5(5.7–7.7)	5.9 ± 0.4 (5.1–6.5)	5.6 ± 0.5 (4.8–6.4)			
	Total	6.7 ± 0.6 (5.7–7.7)	6.2 ± 0.6 (5.1–7.5)	6.0 ± 0.6 (4.8–7.3)			
Abduction ROM (degrees)	Control	100.7 ± 3.4 (97–107)	109.8 ± 2.6 (105–115)	112.8 ± 2.6 (108–118)	<0.001	<0.001	<0.001
	Experimental	100.8 ± 4.2 (94–110)	118.9 ± 4.2 (111–126)	123.2 ± 3.7 (116–128)			
	Total	100.8 ± 3.8 (94–110)	114.5 ± 5.8 (105–126)	118.2 ± 6.2 (108–128)			

Control = control group; Experimental = experimental group; WUSPI = Wheelchair User’s Shoulder Pain Index; ROM = range of motion.

**Table 3:** Effect of time at each treatment level.

		Experimental Group		Control Group	
Outcome	Time	Contrast (95%CI)	*p*-Value	Contrast (95%CI)	*p*-Value
WUSPI	T1 vs. T0	−67.6 (−71.2–−63.9)	<0.001	−39.9 (−43.6–−36.1)	<0.001
	T2 vs. T0	−74.0 (−77.6–−70.4)	<0.001	−43.9 (−47.6–−40.1)	<0.001
	T2 vs. T1	−6.4 (−10.0–−2.8)	0.001	−4.0 (−7.7–−0.3)	0.037
20 m straight line test	T1 vs. T0	−0.8 (−1.0–−0.6)	<0.001	−0.2 (−0.4–0.1)	0.006
	T2 vs. T0	−1.1 (−1.2–−0.9)	<0.001	−0.4 (−0.6–−0.2)	<0.001
	T2 vs. T1	−0.3 (−0.4–−0.1)	0.002	−0.1 (−0.3–−0.1)	0.095
Abduction ROM	T1 vs. T0	18.1 (16.3–19.8)	<0.001	9.1 (7.3–10.9)	<0.001
	T2 vs. T0	22.4 (20.7–24.2)	<0.001	12.1 (10.3–13.9)	<0.001
	T2 vs. T1	4.4 (2.6–6.1)	<0.001	3.0 (1.2–4.8)	0.001

WUSPI = Wheelchair User’s Shoulder Pain Index; ROM = range of motion; CI = confidence interval.

## Data Availability

The datasets used and analyzed during the current study will be made available upon reasonable request to the corresponding author, G.F.
